# Anti-Virulence Activity of 3,3′-Diindolylmethane (DIM): A Bioactive Cruciferous Phytochemical with Accelerated Wound Healing Benefits

**DOI:** 10.3390/pharmaceutics14050967

**Published:** 2022-04-30

**Authors:** Karina Golberg, Victor Markus, Bat-el Kagan, Sigalit Barzanizan, Karin Yaniv, Kerem Teralı, Esti Kramarsky-Winter, Robert S. Marks, Ariel Kushmaro

**Affiliations:** 1 Avram and Stella Goldstein-Goren Department of Biotechnology Engineering, Ben-Gurion University of the Negev, P.O. Box 653, Beer-Sheva 84105, Israel; karingo@post.bgu.ac.il (K.G.); batel.kagan@gmail.com (B.-e.K.); barzasig@post.bgu.ac.il (S.B.); kariny@post.bgu.ac.il (K.Y.); esti.winter@gmail.com (E.K.-W.); rsmarks@bgu.ac.il (R.S.M.); 2 Department of Medical Biochemistry, Faculty of Medicine, Near East University, Nicosia 99138, Cyprus; victor.markus@neu.edu.tr; 3 Department of Medical Biochemistry, Faculty of Medicine, Girne American University, Kyrenia 99428, Cyprus; keremterali@gau.edu.tr; 4 The Ilse Katz Center for Nanoscale Science and Technology, Ben-Gurion University of the Negev, P.O. Box 653, Beer-Sheva 84105, Israel; 5 School of Sustainability and Climate Change, Ben-Gurion University of the Negev, Beer-Sheva 84105, Israel

**Keywords:** antibiotics resistance, quorum sensing, biofilm, phytochemicals, 3,3′-diindolylmethane

## Abstract

Antimicrobial resistance is among the top global health problems with antibacterial resistance currently representing the major threat both in terms of occurrence and complexity. One reason current treatments of bacterial diseases are ineffective is the occurrence of protective and resistant biofilm structures. Phytochemicals are currently being reviewed for newer anti-virulence agents. In the present study, we aimed to investigate the anti-virulence activity of 3,3′-diindolylmethane (DIM), a bioactive cruciferous phytochemical. Using a series of in vitro assays on major Gram-negative pathogens, including transcriptomic analysis, and in vivo porcine wound studies as well as in silico experiments, we show that DIM has anti-biofilm activity. Following DIM treatment, our findings show that biofilm formation of two of the most prioritized bacterial pathogens *Acinetobacter baumannii* and *Pseudomonas aeruginosa* was inhibited respectively by 65% and 70%. Combining the antibiotic tobramycin with DIM enabled a high inhibition (94%) of *P. aeruginosa* biofilm. A DIM-based formulation, evaluated for its wound-healing efficacy on *P. aeruginosa*-infected wounds, showed a reduction in its bacterial bioburden, and wound size. RNA-seq was used to evaluate the molecular mechanism underlying the bacterial response to DIM. The gene expression profile encompassed shifts in virulence and biofilm-associated genes. A network regulation analysis showed the downregulation of 14 virulence-associated super-regulators. Quantitative real-time PCR verified and supported the transcriptomic results. Molecular docking and interaction profiling indicate that DIM can be accommodated in the autoinducer- or DNA-binding pockets of the virulence regulators making multiple non-covalent interactions with the key residues that are involved in ligand binding. DIM treatment prevented biofilm formation and destroyed existing biofilm without affecting microbial death rates. This study provides evidence for bacterial virulence attenuation by DIM.

## 1. Introduction

Resistance to infections, particularly hospital-acquired (nosocomial) infections caused by opportunistic antibiotic-resistant pathogens, leads to increased mortality rates or strengthened severity of patients’ illnesses [1]. Over-dependence on antibiotics has promoted the evolution of multi-resistant pathogens that are highly infectious [2,3], including *Pseudomonas aeruginosa* (common in cystic fibrosis patients and chronic wounds) and *Acinetobacter baumannii*, as well as extended-spectrum β-lactamase producing Enterobacteriaceae [4]. Indeed, Gram-negative bacteria are of particular concern, not only to their prevalence in many resistant infections but also to their extreme efficiency at up-regulating or acquisition of genes encoding for antibiotics resistant pathways, especially when under the selection pressure of antibiotics [5,6,7]. Unfortunately, as increasing numbers of bacterial antibiotic-resistant strains appear, the rate of new antibiotics discovery has stagnated [8].

Treatment failure is associated with the formation of protective and antimicrobial-resistant biofilm structures, in which resident multicellular groupings are embedded in an extracellular polymeric substance (EPS) that gives them 10–1000 times higher antibiotic resistance than planktonic cells [9,10,11,12]. The majority of chronic and recurrent microbial infections occur due to biofilm establishment. Biofilm-associated drug resistance ensures greater bacterial survival producing persistent chronic infections despite intensive antibiotic treatment and host defense mechanisms. This “socialized survival information structure” during infection is based on the mutual communication within the bacterial community rather than on their existence as single, independent units. Indeed, bacteria are highly social organisms that employ a range of sophisticated intercellular communication systems typically known as “quorum sensing” (QS) [13]. A strategy to disrupt biofilms may both help reduce the selective pressure that is typically exerted by conventional antibiotic treatments, and that drives the emergence of resistant bacteria populations [14] and enable better access to antibiotic treatment. Once the biofilm of chronic infections is dilapidated, the track for a complete infection clearance is paved. Therefore, identifying compounds with anti-biofilm properties that are effective, safe, and lack adverse side effects, can provide a solution. When biofilms are destroyed, bacterial resistance to antibiotics is expected to be lowered.

Cruciferous vegetables such as cabbage, cauliflower, broccoli, kale, collard greens, Brussels sprouts, kohlrabi, Savoy cabbage, and gai lan, produce highly active phytochemicals with enhanced clinical benefits, including 3, 3′-diindolylmethane (DIM), a dimeric product of indole-3-carbinol (I3C) [15]. DIM is one of the best-characterized and most abundant bioactive compounds produced from the crucifers family [16]. When raw cruciferous vegetables are chewed or chopped, they release glucosinolate glucobrassicin from the damaged cells which get in contact with the enzyme myrosinase (β-thioglucosidase), localized in intact cells [17,18]. Myrosinase hydrolyzes glucobrassicin to produce glucose and an unstable moiety called thiohydroximate-O-sulfonate which quickly changes into another unstable intermediary molecule (3-indolylmethylisothiocyanate) that in turn splits into I3C and thiocyanate ion [17]. In the low-pH environment of the stomach, I3C polymerizes to form polycyclic aromatic products [19], one of which is DIM. In the case of thermal handling of cruciferous vegetables, myrosinase is inactivated resulting in a reduction of I3C bioavailability [17].

Biological activities and properties of DIM have been the subject of much interest and research. Its many properties, in addition to its prominent chemotherapeutic activity [20], include antiviral, antifungal, antileishmanial [21,22], anti-inflammatory abilities [23], attenuate arthritis, and osteoclastogenesis [24]. This biological versatility led us to investigate the influence of DIM on the formation and destruction of mature biofilms of several pathogenic bacterial strains that are present in chronic infections. Furthermore, since biofilms are key features of chronic wounds, we exploited the wound-associated biofilm model in a porcine to test for the ability of DIM to eliminate biofilms and infection. Managing biofilms have become one of the most important aspects of wound care, therefore, re-purposing highly active and well-studied agent may become a crucial part of the arsenal that specifically address biofilms. This treatment will provide future therapeutic targets for improved chronic wound healing.

## 2. Materials and Methods

### 2.1. Bacterial Strains and Culture Conditions

Four Gram-negative pathogens—*Pseudomonas aeruginosa* PAO1, *Acinetobacter baumannii*, *Serratia marcescens*, and *Providencia stuartii* strains—were grown at 37 °C in LB medium (Difco Luria-Bertani medium, BD, France) with constant agitation at 120 rpm for 24 h. The *E. coli* K802NR strain was cultivated in LB medium supplemented with ampicillin (100 μg mL^−1^) followed by incubation at 30 °C. Overnight inocula were diluted into fresh LB medium to a density of approximately 10^7^ cells mL^−1^ for re-growth at 30 °C to reach an optical density (OD) of 0.2. PAO-JP2 (pKD-*rhlA*), a *lasI-rhlI* double mutant of *P. aeruginosa* PAO1 that harbors pKD vector with *rhlA* promoter coupled upstream to the *luxCDABE* operon. This bioreporter was inoculated into 10 mL LB broth containing 300 µg mL^−1^ trimethoprim and grown overnight at 37 °C with agitation.

### 2.2. Growth Curves Profile

The antimicrobial activities of DIM against *A. baumannii*, *P. aeruginosa* PAO1, *P. stuartii*, and *S. marcescens* were examined by monitoring the growth of these strains. To determine the growth curves for the different Gram-negative pathogens, 2 μL of selected microbial culture at OD = 1 was added to LB media supplemented with 50 µM DIM (dimethyl sulfoxide (DMSO) as solvent). The equivalent volume of the DMSO was used as control. A sample of 200 μL of each combination was incubated with shaking in 96-well plates at 37 °C for 24 h. The turbidity of the medium was measured hourly with a microplate reader (BioTek Instruments, Winooski, VT, USA) at a wavelength of 600 nm.

### 2.3. Biofilm Growth in Static Culture

To assess the potential anti-biofilm properties of DIM in terms of prevention, different strains of bacteria were tested under static growth conditions. Overnight cultures of *A. baumannii*, *S. marcescens*, *P. stuartii*, and *P. aeruginosa* were back-diluted 1:10 in 10% LB medium supplemented with 0.56 mM glucose and 50 µM DIM (DMSO as solvent) or an equivalent amount of the DMSO as the control. All cultures were grown at 37 °C with agitation until they reached the middle exponential phase (OD_600_ ≈ 0.5). Afterward, 1% inoculums were incubated in a glass-bottom 96-well plate (Thermo Fisher, Waltham, MA, USA) under the same conditions as above but supplemented with 50 µM DIM and statically incubated at 37 °C for 24 h. Biofilms were subsequently stained using a LIVE/DEAD *Bac*Light viability staining kit (Molecular Probes Inc., Eugene, OR, USA) according to the manufacturer’s instructions.

### 2.4. Biofilm Growth in Dynamic Culture

To assess the potential anti-biofilm properties of DIM in terms of inhibition and destruction, biofilms were grown in a continuous-culture flow system. For inhibition assessments, biofilms of *A. baumannii* or *P. aeruginosa* PAO1 were grown in three-channel flow cells [25] supplemented continuously with AB trace minimal medium containing 0.3 mM glucose or 10% LB with 0.56 mM glucose for *P. aeruginosa* PAO1 or *A. baumannii*, respectively. After 24 h of growth, the inoculation cultures were back diluted to an OD_595_ of 0.1 in 0.9% NaCl, and 250 µL of the diluted cultures were injected into separate static flow cell channels. After 1 h of incubation, the flow was initiated and maintained at a constant rate of 3 mL h^−1^ at 37 °C using a Masterflex L/S peristaltic pump (Cole-Parmer, Vernon Hills, IL, USA). DIM activity was evaluated by continuously treating one of the channel biofilms with 50 µM DIM supplemented to the medium. For the control, another channel biofilm was treated with an equivalent amount of DMSO. After 48 h under this treatment condition, biofilms were stained, and their thicknesses were measured and assessed using a confocal laser scanning microscope (CLSM) and the IMARIS software (described in more detail separately). For the destruction set of experiments, *P. aeruginosa* PAO1 was cultured in a flow chamber for 72 h until it formed a biofilm, and then 50 µM of DIM and 20 µg mL^−1^ of tobramycin were introduced into the culture for an additional 48 h of treatment.

### 2.5. Antimicrobial Susceptibility Tests

The minimum inhibitory concentration (MIC) values of several antibiotics from among those most prevalently used in CF patients were determined by E-TEST (Biomérieux, Marcy-l'Etoile, France). The antimicrobial concentration ranges of 0.016 to 256 µg/mL were tested for gentamicin, tobramycin, azithromycin, levofloxacin, ciprofloxacin, and colistin. Antimicrobial-agent-coated test strips were placed on 1% Mueller-Hinton agar plates (Himedia Laboratories) supplemented with 50 µM DIM (DMSO as solvent) or an equivalent volume of DMSO and uniformly spread with a lawn of *P. aeruginosa* pre-grown for 24 h in the presence of 50 µM DIM. The MIC value of every antibiotic was assessed according to the manufacturer’s instructions after incubation for 24 h at 37 °C.

### 2.6. Biofilm Visualization

Biofilm architecture and thickness were acquired using an FV1000 CLSM (Olympus, Tokyo, Japan) equipped with a 60 × 1.35NA lens. The excitation wavelength for green-live SYTO 9 dye was 488 nm and emission wavelengths of 515 nm were collected. For the red-dead PI stain, the excitation was 530 nm and emission was recorded at 617 nm. Three-dimensional projections of biofilm structures were reconstructed with quantitative structural parameters of the biofilms using the Easy 3D function of the IMARIS software (Bitplane AG, Zürich, Switzerland).

### 2.7. Qualitative Congo Red Plate Assay of EPS Production

To elucidate the mechanisms of biofilm attenuation by DIM, EPS production, which is directly proportional to the amount of biofilm produced, was assessed. Bacteria were grown on Congo red plates, and EPS was evaluated based on its characteristically black, diffusible pigmentation, as previously described [26]. Briefly, Congo red stain (Sigma, St. Louis, MO, USA) was added at a concentration of 0.8 mg mL^−1^ to a brain heart infusion broth supplemented with 5% sucrose (*w*/*v*), 1% agar, and 100 µM DIM. The centers of the plates were inoculated with drops of *P. stuartii* (2–5 µL) that had been back-diluted 1:10 in 10% LB medium supplemented with each inhibitor (or DMSO for control) and grown for three hours at 37 °C. Plates were incubated aerobically at 37 °C.

### 2.8. Qualitative Assay of Swarming Motility

Swarm agar plates comprised modified M9 minimal medium (62 mM potassium phosphate buffer, pH 7; 7 mM (NH_4_)_2_SO_4_; 2 mM MgSO_4_; 10 µM FeSO_4_; 0.4% (*w*/*v*) glucose; 0.5% casamino acids (Difco)) solidified with 0.5% Bacto-agar (Difco) and supplemented with DIM or DMSO. *A. baumannii* and *P. aeruginosa* PA01 were grown overnight in M9 medium, after which the bacteria were diluted 1:10 into fresh medium supplemented with 100 µM DIM, and incubated at 37 °C with agitation until they reached mid-logarithmic phase (i.e., OD_600_ of 0.4 to 0.6). To assess surface coverage, 2.5 µL of the re-growth bacteria was placed in the middle of each swarm agar plate and incubated at 37 °C for 24 h.

### 2.9. Quantization of Extracellular Virulence Factor Production by P. aeruginosa PA01

A *P. aeruginosa* PAO1 culture grown overnight was diluted in a fresh LB medium (1:600) supplemented with 50 µM DIM and incubated at 37 °C with agitation for 24 h. Virulence factor production by *P. aeruginosa* PAO1 cell-free culture (centrifuged for 15 min at 3700 rpm) was quantified as previously described with minor modifications [27,28]. **Elastase** activity of LasB was assayed using elastin Congo red (ECR) (Sigma). An aliquot of cell-free culture was added in a ratio of 1:3 to ECR buffer (0.1 M Tris-HCl pH 8; 1 mM CaCl_2_) containing 5 mg mL^−1^ ECR, and the solution was incubated at 37 °C with agitation at 200 rpm for one week. The insoluble ECR was removed by centrifugation at 6000 g for 10 min, and absorption of the supernatant was measured at 400 nm. Total **protease** activity of the cell-free culture was determined with 2% azocasein (Sigma) in phosphate buffer saline (PBS, pH 7) in a 1:1 ratio. The resultant reaction mixture was incubated statically at 37 °C for 1 h, and the reaction was terminated by adding 500 μL trichloroacetic acid (10%). The undigested substrate was precipitated by centrifugation at 8000 g for 5 min and assessed by an absorbance reading at 400 nm. **Pyocyanin** was extracted from cell-free culture to chloroform organic phase at a 3:2 ratio. The chloroform phase was transferred to 800 µL of 0.2 M HCl, the upper pink phase was removed and its absorption was determined at 540 nm. **Pyoverdine** was measured by characteristic absorbance at 380 nm. The activity of **chitinase** was assayed by mixing 0.4 mL of cell-free culture with 0.4 mL PBS and 2.5 mg of insoluble chitin-azure (Sigma-Aldrich, Saint Louis, MO, USA). The reaction mixture was then incubated for 24 h at 37 °C with agitation at 200 rpm. After centrifugation (16,000× *g* for 10 min), the insoluble chitin-azure was removed and the absorbance was measured at 570 nm.

### 2.10. Bioluminescence Assay for Anti-QS Activity

QS antagonistic activity of DIM was assessed by comparing the bioluminescence signal observed in wells containing the activated *E. coli* K802NR or *P. aeruginosa* pKD-*rhlA* bioreporter strains carrying the *luxCDABE* operon with that in the wells containing the same reaction mixture but with the addition of 50 µM DIM. Reaction mixtures comprised either *E. coli* K802NR and 1.7 nM *N*-(3-oxododecanoyl)-l-homoserine lactone (3-oxo-C12-HSL) (O9139, SigmaAldrich) or PAO-JP2 (pKD-*rhlA*) and 1 × 10^−5^ M *N*-butyryl-dl-homoserine lactone (C4-HSL) (09945, Sigma-Aldrich). Inhibition was indicated when the addition of DIM to mixtures containing either 3-oxo-C12-HSL or C4-HSL resulted in a reduction in bioluminescence.

### 2.11. RNA Extraction, RNA-Seq, and Transcriptomic Analysis

*P. aeruginosa* PA01 cultures that were grown in the presence of 50 µM DIM or the equivalent volume of DMSO were pretreated with RNAprotect Bacteria reagent (1 mL) (Qiagen, Hilden, Germany). The RNAs were extracted using the RNeasy Mini kit (Qiagen), including on-column digestion with RNase-free DNase I (Qiagen) according to the manufacturer’s instructions. RNA samples were quantified using NanoDrop™ One Spectrophotometer (Thermo Scientific, Waltham, MA, USA) and analyzed for integrity using Agilent 4200 TapeStation. Levels of remaining DNA were checked using Qubit fluorometer (Invitrogen, Waltham, MA, USA). DNA amounts did not exceed 10% of the total amount of nucleic acid.

Illumina sequencing was performed by preparation of one batch library in a 96-well plate using Stranded CORALL total RNAseq library prep kit with RiboCop rRNA. In addition, a Depletion Kit for Gram-negative bacteria (Lexogen) was employed. Briefly, 700–900 ng of total RNA were used for the first rRNA depletion step, then followed by library generation initiated with random oligonucleotide primer hybridization and reverse transcription. No prior RNA fragmentation was done, as the insert size was determined by the proprietary size-restricting method. In the next stage, 3′ ends of first-strand cDNA fragments were ligated with a linker containing Illumina-compatible P5 sequences and unique molecular identifiers (UMIs). During the following steps of second-strand cDNA synthesis and ds cDNA amplification, i7 and i5 indices, as well as complete adapter sequences required for cluster generation, were added. Amplification cycles were determined to be 12 cycles, as determined by qPCR using a small pre-amplification library aliquot for each sample. Final amplified libraries were purified, quantified, and average fragment sizes were confirmed to be 310 bp by gel electrophoresis using TapeStation. Concentration of the final library pool was confirmed by qPCR and then subjected to test sequencing to be able to check sequencing efficiencies and adjust accordingly proportions of individual libraries. Sequencing was carried out on NovaSeq 6000 (illumna), S4 flow-cell, 2 × 150 nt reads.

Raw sequence reads were quality assessed using FASTQC and MultiQC. Quality trimming and filtering of the reads were performed using Trim Galore! The *P. aeruginosa* PAO1 genome sequence and annotation (version ASM676v1) were downloaded from Ensembl. Reads were aligned to the genome using bowtie2 assuming stranded library preparation and quantified using RSEM. Statistical testing for differential expression was carried out using DESeq2. Genes were considered differentially expressed (DE) if they had FDR-adjusted *p*-value < 0.05 and absolute fold change (in linear scale) ≥ 1.3. Hierarchical clustering was done using R’s pheatmap function. Pathway enrichment analyses were performed using PANTHER through David Bioinformatics Resources. The master regulators analysis was performed using the PAGnet R-package of the down-regulated genes. The PAGnet is comprised of 20 master regulators, however, only 14 were affected by DIM treatment.

### 2.12. Reverse Transcription and Real-Time PCR

RNA extractions were performed as described above, and the cDNA was synthesized using One Step PrimeScript RT Master Mix (TAKARA). *P. aeruginosa* PAO1 sequences of *proC* (PA0393), *pilA* (PA4525), *pilG* (PA0408), *fliA* (PA1455), *fliG* (PA1102) and *PA4352*, *lasA* (PA1871), *rhlA* (PA3479) *phzM* (PA4209) genes were identified from GenBank. The primers for the PCR amplification of cDNA were designed using the primer3 program (Table S1). RT-qPCR amplification was executed using Step One Plus real-time PCR 110 system (Applied Biosystems, Thermo Scientific, Waltham, MA, USA). Duplicate PCR reactions were performed using the qPCRBIO SyGreen Blue Mix Hi-ROX (PCR Biosystems, London, UK). Five microliters of a 1:100 dilution of the cDNA were used in a total volume of 20 μL. After a 15 min activation of the modified Taq polymerase at 95 °C, 40 cycles of 15 s at 95 °C and 1 min at 60 °C were performed. A melt curve was run at the end of the 40 cycles to test for the presence of a unique PCR reaction product: 15 s at 95 °C, 1 min at 60 °C, and 15 s at 95 °C.

### 2.13. Formulation of DIM-Based Cream

The cream consisted of only the basic excipients found in the USP RLD formulation of 0.1% gentamicin sulfate cream and the minimum additional excipients required to enhance DIM solubility in the hydrophilic phase of the cream. The hydrophobic phase comprised 10% isopropyl myristate, 5% propylene glycol stearate, 6% polysorbate 40, 0.3% benzyl alcohol, 6% stearic acid, and 0.05% butylparaben. The hydrophilic phase was prepared by mixing 48% water, 8% propylene glycol, 7% sorbitol, and 0.15% methylparaben. While mixing, the hydrophilic phase was slowly added to the oil/surfactant solution. Once the hydrophilic phase was fully incorporated, the cream was homogenized while mixing for 5 min. The inclusion of DIM in the hydrophilic phase was enabled by adding 15% hydroxypropyl-beta-cyclodextrin (final concentration 8%) to the water in addition to 1 g of benzyl alcohol/ethanol (3/7–0.3%/0.7%), the minimum needed to dissolve DIM. Because of the lipophilic nature of DIM, cyclodextrin, a widely used vehicle especially in biofilm-associated infections [29,30] was employed to increase the solubility of DIM and to promote its effective use in pus-excreting wounds. In the total formulation, the final concentrations of DIM and gentamicin were 0.1% and 0.1%, respectively. Overall, we formulated four creams: cream vehicle control, and creams containing 0.1% DIM, 0.1% gentamicin, and 0.1% DIM with 0.1% gentamycin.

### 2.14. Full-Thickness P. aeruginosa-Infected Porcine Excision Wound Model

A pathogen-free female (SPF: Lahav Research Institute (LRI), Kibbutz Lahav, Israel) domestic swine (Crossbreed of Landrace & Large White), weighing 40 ± 5 kg, was acclimated for 5 days. The animal was fed a basal diet and housed individually in a facility with controlled temperature (17–26 °C) and lights (12 h/12 h LD). Administration of the treatments was performed under sedation of 2 mg kg^−1^ Xylazine + 10 mg kg^−1^ ketamine by intramuscular injection, then followed by endotracheal tube isoflurane and oxygen. The flank and back of the animal were clipped with standard animal clippers. The skin on both sides of each animal was prepared for wounding by washing with a non-antibiotic soap and sterile water followed by drying with sterile gauze. Full-thickness skin wounds were made by using a 10 mm punch biopsy. As soon as the bleeding was stopped, the wounds were inoculated with 25 μL 10^8^ CFU mL^−1^ of *P. aeruginosa* PAO1. The infected wounds were immediately covered with a polyurethane film dressing (Tegaderm™ Transparent Dressing; 3 M) and all sites were secured with surgical tape. The entire animal was then loosely wrapped with Coban self-adhesive elastic wrap. The Tegaderm remained glued to the wounds for 48 h to enable the biofilm to form. Treatments were applied in straight lines on the wound sites. The Tegaderm was peeled revealing each treatment line and the treatments were applied by sterile sticks. Five treatment groups—non-treated, vehicle, DIM, gentamicin cream, and DIM plus gentamicin—were tested on the pre-infected wounds (six wounds per treatment). Treatments were topically applied three times a week. Wounds were photographed with a digital camera on days 0, 2, 3, 4, 7, 9, and 10 to assess the progress of wound closure. Ten days post-infection animals were sacrificed, and biopsies were taken to evaluate the bacterial load and the healing process of the wounds relative to group affiliation.

### 2.15. Quantitative Bacteriology

Ten days post-infection, the wound tissues were harvested by 5 mm punch biopsy. To recover bacteria, *P. aeruginosa* infected biofilm wound samples were placed in 1 mL of sterile phosphate buffer saline. Before serial dilutions, the punched tissues were homogenized for 1 min, and the resulting solutions were serially diluted and plated onto Pseudomonas isolation agar (Hylabs) for colony counting. Plates were incubated for 24 h at 37 °C and CFU values were determined.

## 3. Results

### 3.1. Assessing Biofilm Formation of Model Bacteria

The current study investigated the influence of DIM on the biofilm formation process and the destruction of existing biofilms of several pathogenic Gram-negative bacterial strains. The introduction of DIM to bacterial cultures led to the substantial (up to 80%) reduction in biofilm formation of *A. baumannii*, *S. marcescens*, *P. stuartii*, and *P. aeruginosa* PAO1 when compared to the thick, live biofilms of the control samples (Figure S1). “Dead biofilms” are not shown in the micrographs due to their relatively negligible and similar representations between each model biofilm strain and applied treatment. In addition, bacterial growth as measured using kinetic curves showed no effect by the DIM treatment (Figure S2). Treatment with 100 µM DIM under static conditions resulted in the formation of thinner biofilms with *P. stuartii*, *S. marcescens*, *A. baumannii*, and *P. aeruginosa* PAO1 being inhibited by 62%, 82%, 86%, and 76%, respectively (Figure S1).

**Figure 1:**
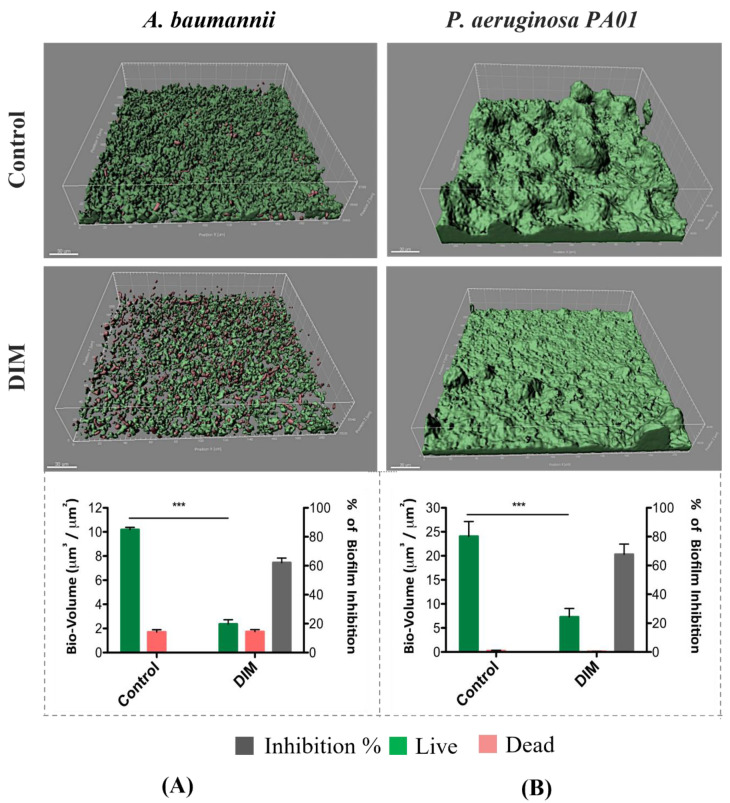
Prevention of the formation of biofilm by *A. baumannii* (**A**) and *P. aeruginosa PA01* (**B**) in a flow system during 48 h of incubation with continuous supplementation of 50 μM DIM. Biofilms were stained with the LIVE/DEAD bacterial viability kit. Quantification of live, dead, and total bio-volumes (μm^3^/μm^2^) based on image analysis and data from the IMARIS software together with % biofilm inhibition. Images were acquired from three different areas in each treatment, three independent repetitions. Asterisks indicate significant differences compared to control (independent samples *t*-test; *** *p* < 0.001).

Considering these data, the prominent activity of DIM against *A. baumannii* and *P. aeruginosa* PAO1 was tested under dynamic conditions, owing to their clinical relevance among multidrug-resistant pathogens. A dynamic growth system, with a continuous flow inlet, was designed to better mimic the actual environmental conditions to which bacteria are exposed. This enabled us to gain valuable insight into the natural development and differentiation processes of biofilm formation [31]. After 48 h of growth, during which both bacterial strains grew thick and dense live biofilms in their respective control channels, morphological differences were observed. *P. aeruginosa* PAO1 formed a characteristic mushroom-like structure when compared to the flatter morphology of *A. baumannii*. The addition of 50 µM DIM to the growth medium led to more scattered and unpacked biofilms (Figure 1), an observation supported by live bio-volume quantification. In the control channels, the live bio-volumes of *A. baumannii* and *P. aeruginosa* PAO1 (10.1 μm^3^/μm^2^ and 24 μm^3^/μm^2^, respectively) were significantly higher than in those under the DIM treatment (2.3 μm^3^/μm^2^ and 7.2 μm^3^/μm^2^, respectively). In this experiment, the overall biofilm inhibition level achieved through exposure to 50 µM DIM was 65% and 70%, respectively (Figure 1). Dead bio-volumes formed during the growth period were similar in terms of volumes.

The slightly stronger effect of DIM on *P. aeruginosa* PAO1 compared to *A. baumannii,* motivated exploration of its anti-biofilm activity in combination with antibiotic treatment. To investigate the combined effects of our biofilm inhibitor compound and a known antibiotic, we cultured *P. aeruginosa* PAO1 for 72 h in the continuous flow system until a mature biofilm was established, and immediately after its establishment, challenged it with 50 µM DIM and/or the antibiotic tobramycin (20 μg mL^−1^). To obtain a better understanding of the impact on bacterial growth observed in the combination treatment, various types of antibiotics were applied in the presence of DIM, revealing different antimicrobial susceptibility patterns (Table S2). Combination with tobramycin was found as the most efficient one with minimum inhibitory concentration (MIC) close to 0.2 µg mL^−1^. Tobramycin was chosen also because it is one of the principal antibiotics of choice in cystic fibrosis (CF) (a biofilm-mediated disease) [32]. Since MIC was established on planktonic cells the concentration for biofilm treatment was set at a value of 100 times more.

**Figure 2:**
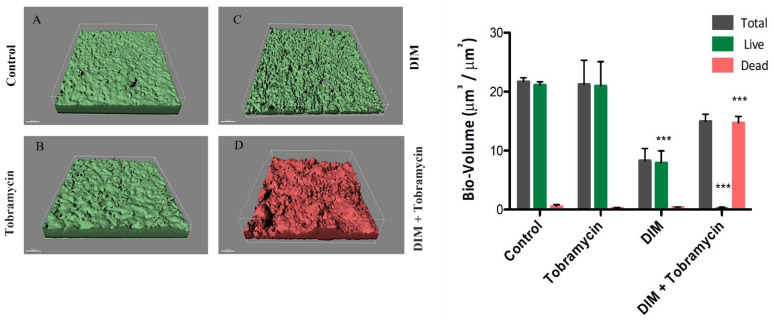
Destruction of mature, differentiated biofilm of *P. aeruginosa* PA01. CLSM images of biofilm formed in a flow system after 120 h (**A**) and continuous supplementation with (**B**) 20 μg mL^−1^ tobramycin, (**C**) 50 μM DIM, and (**D**) a combined treatment of 50 μM DIM with 20 μg mL^−1^ tobramycin, three independent repetitions. Biofilms were stained with the LIVE/DEAD bacterial viability kit. Quantification of bio-volume: live, dead, and total bio-volumes (μm^3^/μm^2^) were calculated based on image analysis and data from the IMARIS software. Differences were analyzed for their significance by using one-way ANOVA with Tukey’s test. *** *p* < 0.0001.

Results of the addition of the effectors alone, or in combination, are as follows: visualization of the biofilms 48 h after the addition of tobramycin showed the formation of a dense biofilm similar to that of the control (Figure 2A,B) and inhibition percentages obtained for this treatment was 1%. DIM alone led to partial destruction of the existing, stable biofilm resulting in a more sparsely distributed architecture with an inhibition percentage of 63% (Figure 2C). This may have possibly occurred due to biofilm detachment from the surface resulting in the planktonic bacteria being washed away. The live bio-volumes of both the tobramycin and the control groups were approximately 20 μm^3^/μm^2^ while that of the thinner and sparser biofilm exposed to the DIM treatment was only 9.3 μm^3^/μm^2^. For all three groups, only minor concentrations of dead cells were evident. In stark contrast, the synergistic DIM-tobramycin treatment almost completely eradicated the biofilm, a difference manifested in the number of dead (red) cells in its CLSM image (Figure 2D) compared to the numbers of live (green) cells in the other images (Figure 2A–C). In line with these observations, statistical analysis indicated that the live bio-volumes were 1.3 μm^3^/μm^2^ while the dead volumes were 17 μm^3^/μm^2^. Indeed, this combination treatment resulted in an inhibition of 98% (Figure 2B–D).

### 3.2. Virulence Factors Attenuation

Optimal QS regulon function in *P. aeruginosa* can be accessed via a comparison between the levels of virulence factors [27] in control vs. DIM treatments. Indeed, the virulence factors that were tested included pyoverdine, a high-affinity Fe(III) chelator that plays a key role in iron acquisition and that mediates signaling [33], the pyocyanin pigment, a factor that chelates the bound iron from transferrin [34], protease, and elastase, which function in concert to destroy host tissues [35], and chitinase, which is believed to break down the chitin-like GlcNAc monomer found in glycoproteins and glycolipids [36]. In the present study we found a significant reduction (43%) in pyocyanin production in the presence of 50 μM DIM, while smaller reductions of 27%, 22%, 10%, and 19% were observed in pyoverdine, chitinase, protease, and elastase, respectively (Figure S3). The activities of pyoverdine and pyocyanin were significantly above the positive control of tetracycline applied at its sub-inhibitory concentration, but for elastase, the opposite trend was found. A strategy that renders pathogenic bacteria non-virulent by interfering with both the *las* and *rhl QS* systems, which jointly regulate the biofilm mode of growth, may therefore facilitate more effective pathogen clearance by the immune system. This notion was further supported by the observation that the addition of DIM to the activated QS bioreporter of either K802NR or PAO-JP2 (pKD-*rhlA*) reduced the bioluminescence output signal of the bioreporters (Figure S4). Using this assay, we showed that 50 µM DIM inhibited both *rhl* and *las* QS systems.

### 3.3. Mechanisms Underlying the Anti-Pathogenic Activity

Based on the assays run on various model bacterial strains, we concluded that DIM may be a potential anti-biofilm agent, interfering with several stages of biofilm formation. To begin with, the equilibrium between attachment to and detachment from the surface is allegedly disturbed by the enhanced swarming motility observed in *P. aeruginosa* PAO1 and *A. baumannii* in the presence of DIM. As shown in Figure S5, colonies of the two model strains that were tested expanded characteristically from the center outward. The addition of DIM led to greater expansion radii, suggesting the planktonic cells could have reduced ability to adhere to a surface. DIM was also assessed for its ability to attenuate the EPS production of all the tested pathogens. Owing to the specificity of the assay dictated by the unique composition of EPS, a positive response was obtained only for *P. stuartii* (Figure S5), the biofilm of which was also found to be attenuated by DIM (Figure S1). Quantification of the EPS relied on the extent of black pigment formation, which directly correlated with the levels of Congo red bound to exopolysaccharides, including glucose-rich polymers [37]. The inhibition of this ability, as evident in the Congo red plate assays of *P. stuartii*, weakens bacterial surface adherence and resistance to antimicrobials.

**Figure 3:**
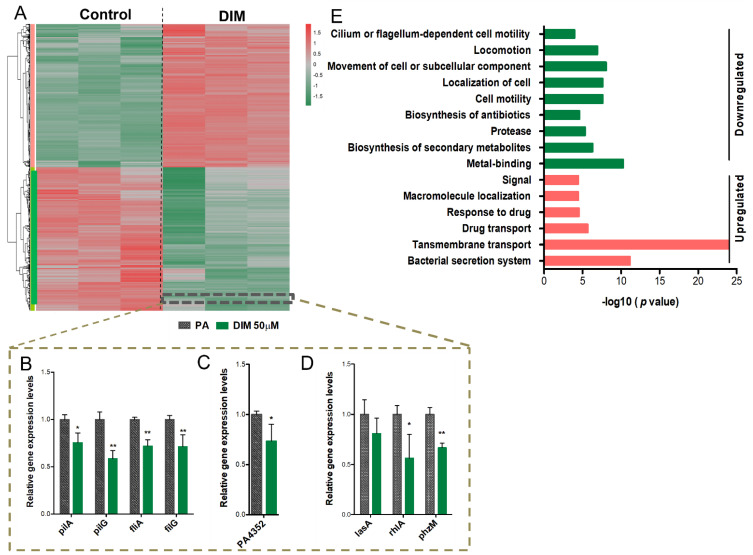
Heterogeneity in gene expression of *P. aeruginosa* PA01 in response to 50 µM DIM. Hierarchical clustering and heat map of RNA-seq data (**A**). Counts were normalized with DeSeq2 and then transformed via log 2 prior to clustering and visualization. Genes were considered differentially expressed if they had FDR-adjusted *p*-value < 0.05 and absolute fold change (in linear scale) ≥ 1.3. Downregulation of a gene is indicated in green, and upregulation is indicated in red. The dashed box denotes genes whose expression was further verified by RT−PCT: motility−associated genes (**B**), stress response gene (**C**), and virulence encoding factors (**D**). The relative magnitude of gene expression level was defined as the copy number of cDNA of each gene normalized by the copy number of cDNA of the housekeeping gene, proC. Error bars indicate the SD of at least three measurements. ** *p* < 0.005 vs the control. * *p* < 0.05 vs control. Genes that were differentially expressed (log 2fold change (FC) ≥ 1.3) in the transcriptional profile were assigned to David’s functional annotation chart (combined heterogeneous annotations). The enrichment of functional categories representing both downregulated pathways (green) and upregulated ones (red) (**E**). Categories with significantly enriched genes are depicted as FDR < 0.05 and *p*-value ≤ 0.05, while log10 (*p*-value) is indicated at the x-axis.

Transcriptomic analysis using NGS technology was conducted to elucidate the mechanism through which DIM exerts its ant-virulence and anti-biofilm activities on *P. aeruginosa*. Statistical analysis was conducted using the DeSeq2 R package. Of the 5571 genes that comprise the *P. aeruginosa* genome, 4073 were found to be significantly and differentially expressed (adjusted *p*-value ≤ 0.05 and FC ≥ 1.3). These genes were then submitted to hierarchical clustering, and subsequent exposure of *P. aeruginosa* to DIM treatment resulted in pronounced differences in the gene expression patterns when compared with that of the control (Figure 3A). A clear and remarkable separation was observed, 2034 genes showed significant upregulation (>1.3-fold), and 2039 genes were significantly downregulated (>1.3-fold). Among the downregulated genes, 40 are found to be involved in cell motility including type IV pilus biogenesis (pil), flagellar (flg), flagellar motor (fli), flagellar apparatus (flh), and chemotaxis (che) genes. The expression of hydrogen cyanide (HCN) encoded by *hcnABC* operon that contributes to high mortality rates during infection of the host [38] was reduced. Likewise, the virulence cytotoxic factor phenazine pyocyanin which substitutes the greenish pigment of sputum and pus in *P. aeruginosa*-associated infections (*phzABCDEFG*, *phzM*, *phzS*) [39] was extensively reduced. A marked decrease in the transcription of mediating iron acquisition operons by two major siderophores, pyoverdine (Pvd) and pyochelin (Pch), and iron storage heme-containing bacterioferritins (Bfr) have also been seen. These siderophores possess dual activity. On the one hand, *P. aeruginosa* utilizes them to scavenge host iron, a key nutrient needed for its growth. On the other hand, they act as a signaling molecule for the production of other virulence factors [40]. DIM also repressed the expression of virulent factors that are encoded by a single gene setup including elastase (*lasB*), protease (*lasA*), alkaline protease (*aprA*), rhamnolipids (*rhlA* and *rhlB*), and lectin (*lecA*). Compounding all these findings, we propose that DIM may turn down *P. aeruginosa* virulence behavior that encompasses arsenal factors and biofilm fortress, and by that affect the pathogenicity pathway as shown both by the transcriptomics analysis and virulence factors quantification (Figure S3).

Furthermore, qRT-PCR was used to confirm the RNA-seq results of selected genes. Genes associated with motility (*pilA*, *pilG*, *fliA,* and *fliG*), survival protein PA4352, a member of a universal stress protein (Usp) [41], and virulence factors encoding genes (*rhlA* and *phzM*) were all significantly downregulated (Figure 3B–D). To better understand the biological functions and the metabolic pathways of the identified genes, the DEGs were functionally categorized based on the clusters of orthologous groups (COGs) using Database for Annotation, Visualization, and Integrated Discovery (DAVID) of different annotation databases. Expression of genes associated with transmembrane transport and drug secretion were upregulated while genes with downregulated expression include those associated with cell motility, antibiotic biosynthesis, proteases virulence response, and metal ion chelators (Figure 3E). These pathways may infer the possible mechanisms underlying the changes in gene expression. To comprehensively investigate the effect of DIM on master-regulators associated with virulence regulatory pathways such as QS, T3SS, T6SS, and biofilms, we have employed *P. aeruginosa* Genomic regulatory network (PAGnet) [42].

**Figure 4:**
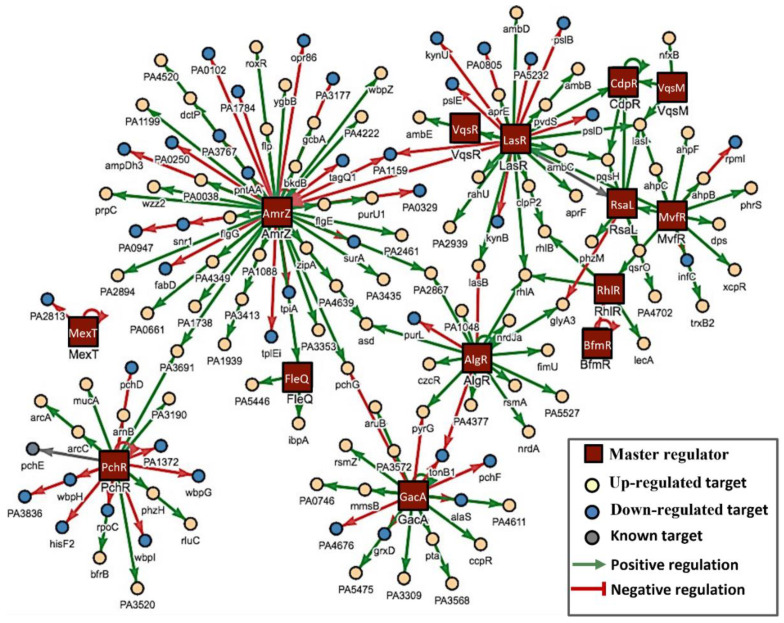
PAGnet master regulators network. The regulons shown as red squares represent 14 downregulated master regulators of *P. aeruginosa*. The red solid line is the negative regulation, and the green solid line is the positive regulation. The individual circle presents the functional targets of each regulon. Positively regulated targets (yellow), negatively regulated targets (blue).

Out of 20 key regulators, 14 were downregulated when *P. aeruginosa* was exposed to DIM (Figure 4). Our regulatory network can be categorized according to functionality including QS regulators (RsaL, RhlR, CpdR, MvfR, PchR, and LasR), biofilm formation (BfmR, AmrZ, GacA, FleQ, LasR, MvfR, RhlR, and VqsM), T6SS (AmrZ) and T3SS (MexT and VqsM).

### 3.4. In Silico Studies

**Figure 5:**
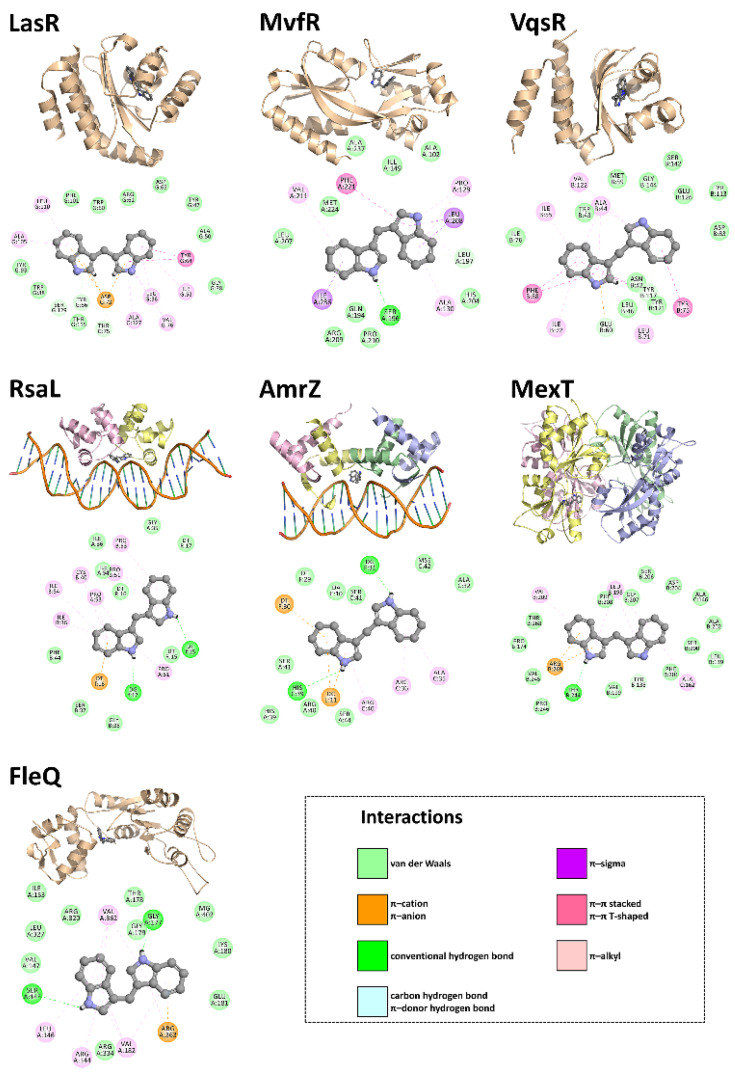
Structural representations of the best binding poses of DIM against *P. aeruginosa* targets. Fully automated protein–ligand docking was achieved via JAMDA, which combines the TrixX docking algorithm [43,44] with the JAMDA scoring function [45]. For LasR, MvfR, and VqsR, the docking search space was confined to the autoinducer-binding pocket in the PDB entries 2UV0 [46], 6Q7U [47], and 5XHX [48], respectively. For RsaL and AmrZ, DoGSiteScorer [49] was used to detect the relevant binding pocket in the PDB entries 5J2Y [50] and 3QOQ [51], respectively. For MexT, the docking site was defined by a sulfate ion occupying the putative ligand-binding pocket in the PDB entry 6L33 [52]. For FleQ, the docking search space was confined to the ATP-binding pocket in the PDB entry 6J7E [53]. Images of the predicted virulence regulator–DIM complexes were rendered using the PyMOL Molecular Graphics System, Version 1.8.6 (Schrödinger LLC, Portland, OR, USA). A single RsaL dimer was created by implementing the Symmetry Mates utility program in PyMOL. Non-covalent interactions between the *P. aeruginosa* virulence regulators and DIM were estimated using Discovery Studio Visualizer, Version 16.1.0 (Dassault Systèmes BIOVIA Corp., San Diego, CA, USA).

It could be that DIM directly interacts with these virulence-related transcription factors, either causing their rapid turnover or interfering with their target gene binding/transactivation function. To test this hypothesis, we docked DIM onto *P. aeruginosa* virulence-related transcription factors with resolved crystal structures in their ligand-bound conformations. The ligand was a small molecule (e.g., AHL) in some structures, while it was DNA in others. Our results indicate that DIM can be accommodated in the autoinducer- or DNA-binding pockets of the virulence regulators in question in an energetically favorable manner—i.e., with increasingly negative docking scores—and that it can establish multiple simultaneous non-covalent interactions with the key residues that are involved in ligand binding (Figure 5).

### 3.5. Dermal Excisional Wound Healing in a Porcine Model Following DIM Treatment

**Figure 6:**
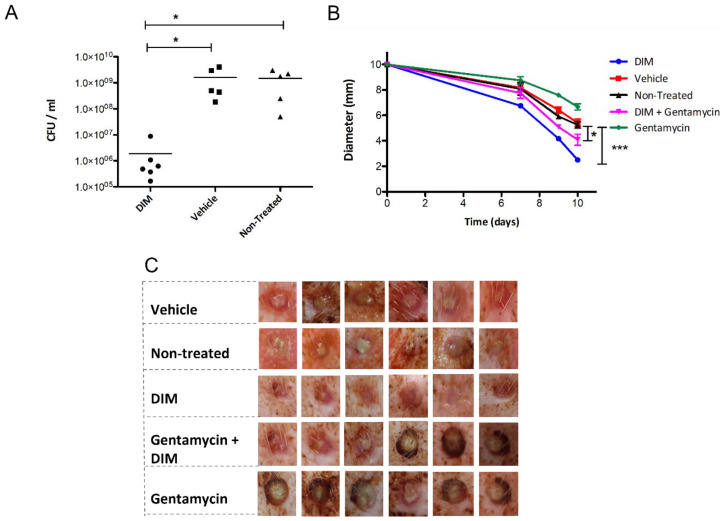
Quantification of *P. aeruginosa* PA01 strain isolated from the wound-associated biofilm. Biofilms were allowed to develop for 48 h before the formulated cream treatments were applied. (**A**) Punch biopsies were collected on day 10 post-infection, and the CFU mL^−1^ was determined. Asterisks indicate significant differences when compared to vehicle or non-treated groups (independent samples *t*-test; * *p* < 0.05; *** *p* < 0.001). Wound healing in porcine model infected with *P. aeruginosa* PA01. (**B**) Mean diameters of the non-treated wound and wounds treated with DIM, gentamycin, vehicle, and a DIM-gentamycin combination on day 10. Values are the averages of the results for six measurements and error bars denote standard deviations. Student *t*-test calculated *p*-values of <0.05 were considered significant. (**C**) Healing of 10-mm diameter, full-thickness excision wounds on the back of domestic swine (crossbreed of Landrace & Large White genetic strains) was monitored using digital photography. Representative photos taken on day 10 are shown. Data are reported in mean scores ± SD (*).

As it has been suggested that the failure of chronic wounds to heal is mainly due to the presence of *P. aeruginosa* biofilms [54], we tested this conjecture in a porcine wound model with a DIM challenge. We performed that as a preliminary test of concept with further extensive studies on the way in that direction. Two-day-old *P. aeruginosa* PAO1 biofilms were established in the porcine full-thickness wounds by direct inoculation with an average start inoculum of 3 × 10^8^ CFU mL^−1^, after which the wound was covered with a polyurethane film dressing. The infected wounds developed herein showed the characteristic phenotype of *P. aeruginosa* biofilm, i.e., viscous greenish pus excretions. Creams formulated with DIM and/or gentamycin were topically applied at five time points during the ten days following the establishment of the model. A comparison of the bacterial loads of the wound models after 10 days showed that the highest efficacy was obtained for the treatment with DIM alone. The significantly lower bacterial counts observed were approximately three-fold lower than those obtained for the vehicle or non-treated controls (Figure 6A). Since tobramycin (used mostly as an ophthalmic medication) is not prescribed to treat topical infection, gentamicin, being a broad-spectrum aminoglycoside widely used to treat a variety of skin infections, was selected. The antimicrobial efficacy of gentamycin was markedly impaired in the biofilm-treated wounds relative to that of the DIM treatment alone, thus fulfilling a criterion for biofilm-associated disease, i.e., delayed wound recovery. Groups treated with DIM and with the combined treatment of DIM with gentamycin exhibited significantly decreased wound sizes (Figure 6C). Wound diameters, initially 10 mm, were monitored during the 10-day experiment and measured on days 7, 9, and 10 (Figure 6B). Treatment with the DIM compound alone, which was effective against infected wounds, enhanced and accelerated the healing process. The final wound diameters were 2 mm. The observed decrease in wound size indicating faster healing was significant compared to that obtained for the vehicle control group. The gentamycin treatment, not only failed to promote wound closure or contraction but also slowed the healing process and formed scabbed lesions that prevented the closure of the skin margins (Figure 6C). Wounds in the non-treated groups did not show any change, indicating that the vehicle by itself lacks any healing capacity. On day 10, the wounds treated with DIM were almost healed relative to the controls or antibiotic groups. Overall, when used alone, DIM was found to accelerate the healing process better than antibiotics or DIM-antibiotic combination.

## 4. Discussion

The ubiquity of the bacterial biofilm and its exploitation by numerous bacteria as a mode of survival in inhospitable environments has wide-ranging and significant impacts on human life. Of particular concern, are the clinical threats posed by multi-drug-resistant opportunistic pathogens. Biofilms affect the disease-causing ability of pathogens thus causing potential health issues of machine and tool surfaces in clinical settings. This increase in biofilm-related infections has become a key driving force behind efforts to find novel methods to inhibit biofilm growth or to cause the breakdown of existing biofilms. Drug repurposing, involving the identification of new applications of an approved medication beyond the scope of its original specification, is gaining considerable attention in recent years. We show here that the indole derivative DIM has anti-biofilm activity. DIM showed a marked inhibitory activity against the biofilm formation of our bacterial pathogenic models.

Due to its various biological activities in different bacterial strains, indole has drawn considerable attention among bacterial signal molecules. Indole, produced from tryptophan by a wide range of Gram-positive and Gram-negative bacterial species, is sensed via their membrane-bound histidine sensor kinase (HK) CpxA [55] and regulates a variety of processes that give enormous advantages to these bacteria [56]. Indole signaling is dynamic, having impacts on bacteria differently—advantageous (to indole producers) and deleterious (to most non-indole producers). Indole has been shown to inhibit acyl-homoserine lactone (AHL)-dependent quorum signaling [57,58]. Natural agents having DIM units such as marine bisindole alkaloid 2,2-bis(6-bromo-3-indolyl)ethylamine, produced by tunicate and sponge, have been shown to possess anti-biofilm activity [59]. Together with our findings, these reports indicate the potential of indole derivatives as anti-virulence molecules. The plant-derived indole, I3C, has been shown to bind to the aromatic hydrocarbon receptor (AhR) found in most tissues, including the gut [60] where it decreases colitis by reducing microbial dysbiosis and boosting the abundance of butyrate-producing Gram-positive bacteria in an IL-22–dependent way [61]. Serval bacteria and eukaryotes including humans that do not synthesize indole encode enzymes that can modify/degrade the indole into derivative products [60,62,63].

Drug-resistant biofilm-forming bacteria are known to pose an even greater threat once they have already infected and damaged healthy tissue, therefore the addition of an anti-biofilm compound to traditional antibiotic treatments may improve treatment outcomes. Indeed, the combination of the antibiotic tobramycin with DIM gave a higher biofilm inhibition than DIM alone, suggesting that using combination therapy that includes DIM may salvage antibiotic potency via said synergy. The sparsely distributed biofilm observed following DIM treatment suggests a connection between several super regulators associated with biofilm formation. The disruption in bacterial biofilms affected by their exposure to DIM leads to the development of unstable and weak biofilm structures, ultimately resulting in biofilm dispersal. Considering our results, DIM alone showed effectiveness; however, the finding of an unexpectedly large bio-volume following the combined treatment is not quite clear. We hypothesized that this may have occurred by the synergistic DIM-tobramycin activity in inducing a stress response that is manifested by a thicker biofilm. DIM, however, was able to restore the efficacy of tobramycin without affecting the biofilm dispersal stage. The inability of tobramycin alone to inhibit or destroy differentiated biofilm is ascribed to the bacterial multifactorial tolerance mechanism [64]. The most prominent mechanisms involved in this tolerance response include, among others, restricted antimicrobial diffusion, differential physiological activity, induction of specific tolerance mechanisms, and metabolically dormant “persister” cells [65]. In addition, the antibiotics may also be deactivated by specific biofilm-associated factors involved in maintaining the resistance of the bacterial cells within the biofilm. Concrete activity against tobramycin was demonstrated by glucose polymers in the form of periplasmic glucans found to be produced only in the embedded biofilm cells. These polymers effectively sequestered the antibiotic in the periplasmic space by direct binding, thus preventing it from reaching its target within the bacterial cells in a process that ultimately protects the entire community [66]. The effectiveness of the DIM-tobramycin combination treatment, observed in this study, is encouraging, considering the progressive increase in the number of bacterial species that exhibit antibiotic resistance in this “post-antibiotic era”. The emergence of severe bacterial infections is believed to result primarily from the evolution of “superbugs” that possess resistance to a wide range of commonly prescribed antibiotics. Such resistant bacterial infections are becoming considerably more expensive to diagnose and treat because they necessitate longer treatment times, the administration of chosen antibiotics at higher dosages, or even the administration of several antibiotics at once [67].

To bypass the selection pressure imposed by the broad-spectrum antibiotics that have, to date, been the backbone of our conventional antimicrobial arsenal [67,68], advanced anti-pathogen drugs devoid of any bactericidal activity are being developed. From the perspective of this novel strategy, components of these combinations should, like what occurs in the DIM treatment, not inhibit growth, or exert selection pressures while also not affecting normal micro-flora. In a study published by Roy et al. [69], various DIM derivatives were synthesized and evaluated in terms of their antibacterial activity against several bacterial strains. The MIC value of DIM among Gram-negative bacteria such as *P. aeruginosa* was 1000 µM, and the concentrations of most of the derivatives tested by Roy and colleagues [69] ranged from 500 to 2000 µM. These findings support our observations that in both types of biofilm growth experiments, treatment with DIM at concentrations considered to be within the non-toxic therapeutic range (only 50–100 µM) prevented biofilm formation and destroyed existing biofilm without affecting microbial death rates. Overall, this finding supports the potential therapeutic applications of DIM and should be examined in extensive preclinical and clinical studies.

To better understand the mechanism of action behind the biofilm dispersal activity of DIM, motility behavior of the colonies was assessed. Colonies of our model bacteria were shown to have greater expansion radii following the addition of DIM. Such intensified chemotaxis ultimately harms the ability of planktonic cells to adhere to a surface in the first place, thus inhibiting the formation of the initial micro-colonies that constitute the basis of imminent, stable slime communities. This finding is in line with a previous report that the transition from reversible to irreversible attachment during biofilm formation in the hyper-swarming *P. aeruginosa sadC* mutant is defective [70]. Another stage of biofilm formation in which DIM appears to affect is that of EPS production, a crucial step for bacteria to gain resistance and permanence within the biofilm structure. Indeed, EPS fulfills several important functions in both the establishment and maintenance processes of the biofilm, including its mechanical stability, the mediation of bacterial adhesion to surfaces, and the formation of the structural polymeric network that interconnects the embedded cells [71]. Flagella assembly pathway, which mediates swimming and twitching motility, is essential for biofilm development [72]. DIM affected not only the movement ability but also the virulence potency of *P. aeruginosa* that enable rapid adaptations to a wide range of clinical niches. Reduction in the expression of a variety of virulence factors and extracellular degradative enzymes was observed following DIM treatment. The results of our in silico experiments demonstrate that DIM can be housed well in the autoinducer- or DNA-binding cavities of the virulence regulators of interest and that it can simultaneously form sufficient non-covalent interactions with the surrounding active-site residues that are involved in ligand binding, possibly owing to its relatively small size and particular chemical make-up.

Findings that both biofilm formation and bacterial virulence of numerous clinically relevant bacteria are subject to complex regulation networks indicate that one promising anti-biofilm strategy may be the attenuation of bacterial communication circuits using an approach that effectively targets the biofilm without affecting bacterial growth [73]. Such a strategy may reduce resistance, as it eliminates the selective pressure that is typically exerted by conventional antibiotic treatments, which in turn drives the emergence of resistant bacteria populations [14]. The virulence factors of *P. aeruginosa* are produced by its hierarchically interconnected communication system, in which the *las* system dominates that of *rhl* [74,75]. Therefore, virulence factors such as elastase and protease, regulated for the most part by the *las* system [35,75,76] are expressed before those controlled by the *rhl* system, e.g., those that lead to pyocyanin, pyoverdine, and chitinase production [13,35]. This sequential regulation of QS-associated genes enables bacteria to transcribe the different virulence determinants specific to each infection stage [77]. The basis of pathogenicity of clinically relevant bacteria such as *P. aeruginosa* is an arsenal of virulence factors designed for survival and proliferation in the host, enabling invasion and the subsequent establishment of infection. In our study, we compare the levels of several virulence factors [27] in control vs DIM treatments. The results show a significant reduction in pyocyanin production and slight reduction in pyoverdine, chitinase, protease, and elastase, in the presence of 50 μM DIM. Since the production of the virulence factors depends on quorum-sensing (QS) system regulated by a QS regulon [78], the optimal QS regulon function in *P. aeruginosa* was assessed. DIM reduced the bioluminescence output signal of the recombinant bioreporters. The *rhlA*-based C4-HSL bioreporter is transcriptionally activated in response to the presence of a short-chain AHL, e.g., C4-HSL [79] while K802NR bioreporter carries the pSB1075 plasmid that contains a fusion of *lasRI* in which the *lasI* promoter is transcriptionally activated in response to the presence of long-chain AHLs, e.g., 3-oxo-C12-HSL [80]. DIM may have interfered with C4-HSL and 3-oxo-C12-HSL, which are required for cognate receptor binding.

It is widely acknowledged that biofilms constitute numerous chronic infections, delaying the healing process of chronic wounds. To test the efficacy of the repurposed DIM molecule, porcine wound model was employed since it is a close approximation of human skin wounds. In chronic wounds, over 90% of bacteria are embedded in a biofilm matrix [81]. Impaired healing in chronic wounds is attributed mainly to the presence of bacteria in the form of biofilms that promote the establishment of persistent infections and that help the bacteria evade host immunity [82].

Owing to their excellent biocompatibility, biostability with biological fluids and cells, and abundance in nature, plant-based materials have found biomedical applications. In tissue engineering and regenerative medicine, they have been used as an excellent option for developing and creating innovative medical textile and nanoarchitecture tissue scaffolds and grafts [83]. Studies have shown that plant polymers, particularly carbohydrates, made into gel systems, serve as drug carriers for a variety of medicinal applications [84,85,86,87,88]. Besides their use as drug carriers, these carbohydrate polymers serve also as reporters that monitor drug delivery performance [84]. Other plant-based materials such as chiral protein superparticles now show promises in immunotherapy and tumor suppression [89]. Additionally, secondary plant metabolites like polyphenols, alkaloids, flavonoids, and stilbenoids are currently utilized as interventions in a variety of neurological, immunological, and metabolic diseases and as foods to boost health in various parts of the world [90]. Although used for a long time in traditional medicine, secondary plant metabolites are now being explored and studied for their anti-bacterial properties owing to the growing antibiotic resistance by pathogens. Our study underpins the potential of phytochemicals, currently being reviewed for newer antibacterial agents.

The most significant result of the present study is the novel anti-biofilm activity found for DIM against different Gram-negative bacteria. We showed that DIM can be employed in combinational therapy with antibiotics to enhance the potential of the latter to treat drug-resistant bacteria. This indicates a potentially promising strategy for the eradication of biofilm complexes in medical settings. Moreover, this approach could also reduce the prevalence of nosocomial infections. Our results indicate that the observed effects of DIM together with antibiotics have an additive value when combating drug-resistant opportunistic bacteria through anti-biofilm strategies. The possible effects of these combinations on humans are at the core of future investigation of this inhibitor as a potential anti-biofilm therapeutic candidate. Moreover, since the chemopreventive effect of DIM (e.g., cancer) has already been observed in preclinical in vitro and in vivo studies, and research has progressed to the clinical trial phase [15], this compound may likely have an important and definitive role in treating a wide range of human diseases, including the ones caused by drug-resistant biofilm-forming bacteria. Although DIM has been shown to effectively limit larval settlement, providing an antifoulant effect in the marine environment [91], to the best of our knowledge, our study is the first to show the anti-biofilm activity and virulence attenuation of the agent against Gram-negative bacteria. Moreover, a recent study showed that DIM has antibiofilm activity on *Candida albicans* and acne-causing bacteria [92]. Based on its anti-biofilm properties, which affect diverse strains of bacteria, the results of this study suggest that DIM is potentially suitable for attenuating a wide range of bacterial infections. A 10-day treatment of infected full-thickness wounds in porcine, of which the DIM treatment was the most effective, showed complete knockdown of mature *P. aeruginosa* biofilm and augmented healing. Further explorations of such novel applications are needed in terms of dosage and toxicity, resistance gaining, and methods developments in evaluating the success of this treatment strategy. Such studies will reveal the extent to which this novel approach can be exploited to prevent, inhibit, and destroy the ability of infectious bacteria to form biofilms, and as such, bring us closer to eliminating biofilm-related infections.

## Data Availability

Not applicable.

## Supplementary Materials

The following supporting information can be downloaded at: https://www.mdpi.com/article/10.3390/pharmaceutics14050967/s1. Figure S1: Attenuation of biofilms produced by the pathogens *P. stuartii*, *S. marcescens*, *A. baumannii,* and *P. aeruginosa* PAO1. Figure S2: Growth profiles of strains. Figure S3: Inhibition of virulence factor production in *P. aeruginosa* PAO1 that was grown in the presence of 50 μM DIM or 0.6 µg mL^−1^ tetracycline treatment as a positive control. Figure S4: Effect of DIM on bioluminescence induction. Figure S5: Qualitative assays of *P. aeruginosa* PAO1 and *A. baumannii* swarming motility. Table S1: Primer sequences. Table S2: Minimal inhibitory concentrations (MICs) of six antibiotics in µg/ml against *P. aeruginosa* PAOmucA22 Ref [93] is mentioned in the Supplementary Materials.
