# Supplementary material for: Anti-Virulence Activity of 3,3′-Diindolylmethane (DIM): A Bioactive Cruciferous Phytochemical with Accelerated Wound Healing Benefits
Source: Pharmaceutics. 2022 Apr 30;14(5):967. doi: 10.3390/pharmaceutics14050967 (PMC9144697; doi:10.3390/pharmaceutics14050967)
Supplement: Supplementary file 1 [file pharmaceutics-14-00967-s001.zip › pharmaceutics-1675513-supplementary.pdf]

pharmaceutics

MDPI

Supplementary Materials: Anti-infective activity of 3,3′-diindolylmethane (DIM): a bioactive cruciferous phytochemical with accelerated wound healing benefits

Karina Golberg, Victor Markus, Bat-el Kagan, Sigalit Barzanizan, Karin Yaniv, Kerem Teralı, Esti Kramarsky-Winter, Robert S. Marks and Ariel Kushmaro

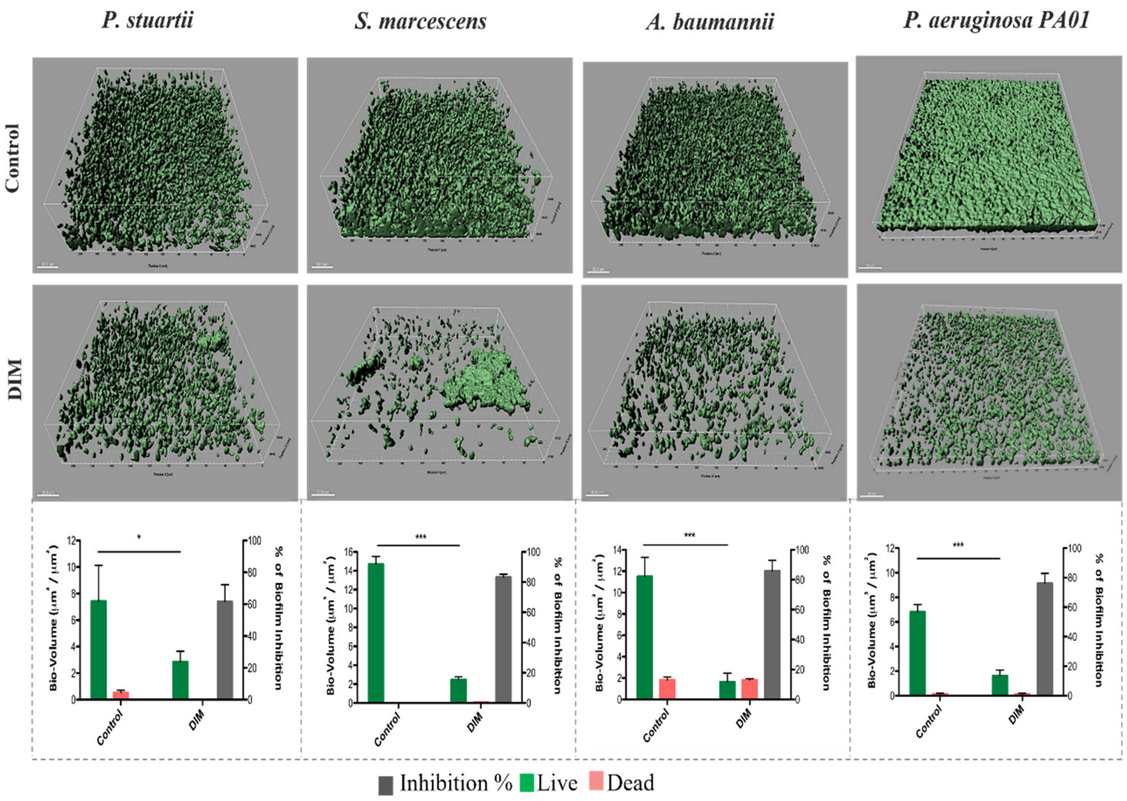

Figure S1. Attenuation of biofilms produced by the pathogens *P. stuartii, S. marcescens, A. baumannii,* and *P. aeruginosa* PAO1. Representative confocal laser scanning microscope (CLSM) images of live biofilms formed in glass-bottom 96-well plates after 24 h of static incubation. Cultures were grown in the presence of either 100 µM DIM or an equivalent amount of DMSO for control. Biofilms were stained with the LIVE/DEAD bacterial viability kit. For each bacterium and treatment, image analysis and data from the IMARIS software were used to calculate live, dead, and total bio-volumes ($\mu m^3/\mu m^2$), and % biofilm inhibition was calculated based on live bio-volumes. The graphically presented results show the average values of at least three micrographs. Those that differed significantly from the control based on a two-tailed *t*-test are labeled with * $P < 0.05$; *** $P < 0.0001$.

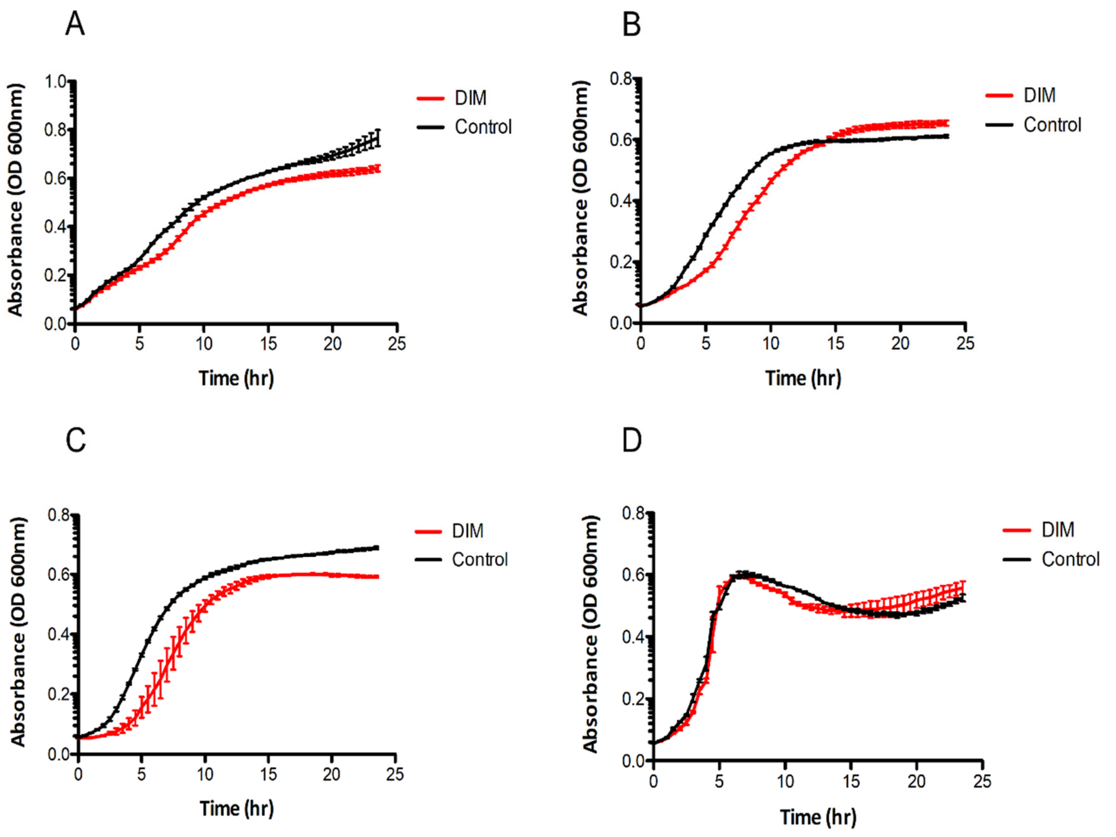

Figure S2. Growth profiles of strains. *S. marcescens* (A), *P. stuartii* (B), *A. baumannii* (C), and *P. aeruginosa* PAO1 (D). The strains were grown in the presence of either 50 µM DIM or an equivalent amount of the solvent. Growth was monitored at OD_{600}. Curves shown are representative of three independent experiments.

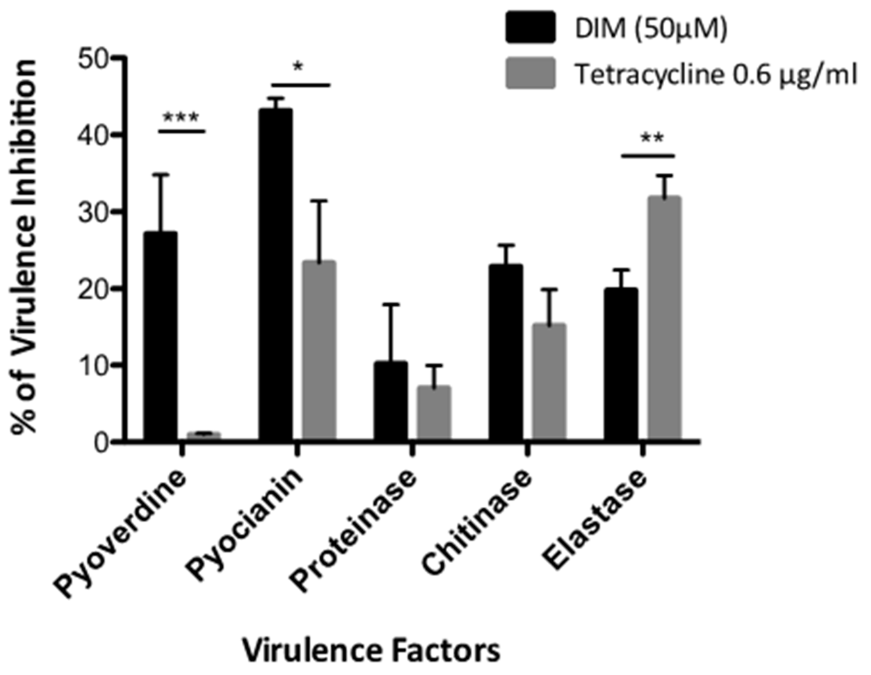

Figure S3. Inhibition of virulence factor production in *P. aeruginosa* PAO1 that was grown in the presence of 50 µM DIM or 0.6 µg mL^{-1} tetracycline treatment as a positive control (the percentage was calculated relative to the negative control,

DMSO). Results are based on OD measurements specific to each factor and normalized to the growth OD of 600 nm. Bars indicate standard deviations for triplicate sets of experiments. Asterisks indicate significant differences when compared to tetracycline (independent samples *t*-test; $^*P < 0.05$; $^{**}P < 0.01$; $^{***}P < 0.001$).

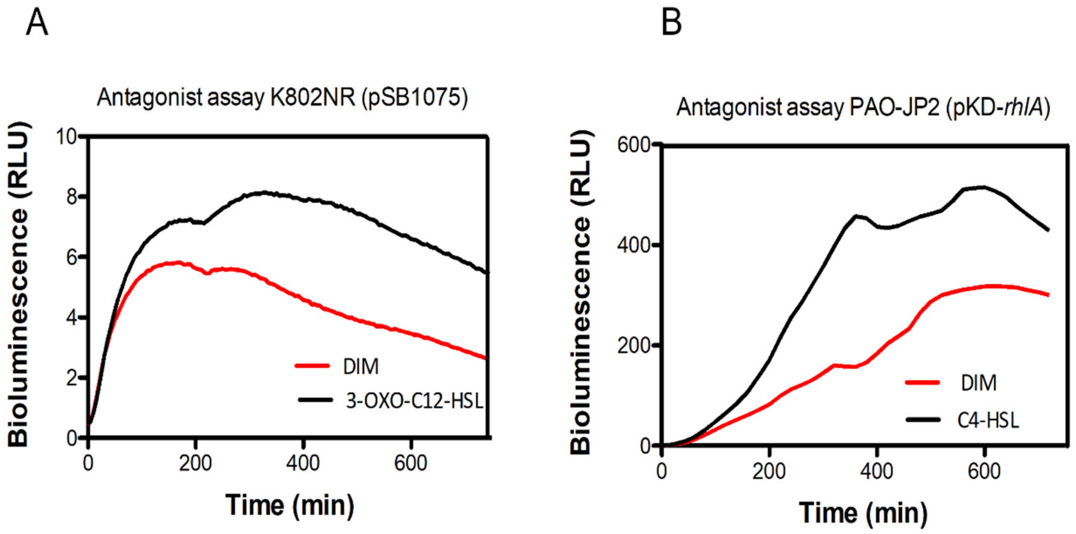

Figure S4. Effect of DIM on bioluminescence induction. Bioluminescence measurements (RLU) over time of reporter strains *E. coli* K802NR (pSB1075) (A) and PAO-JP2 (pKD-*rhlA*) (B) carrying the *luxCDABE* (luciferase) operon in the presence of 50 μM DIM and with exogenous induction by *N*-(3-oxododecanoyl)-L-homoserine lactone (3-oxo-C12-HSL) (2×10^{-8} M) and *N*-butyryl-DL-homoserine lactone (C4-HSL) (1×10^{-5} M), respectively. Values represent means ($n = 4$).

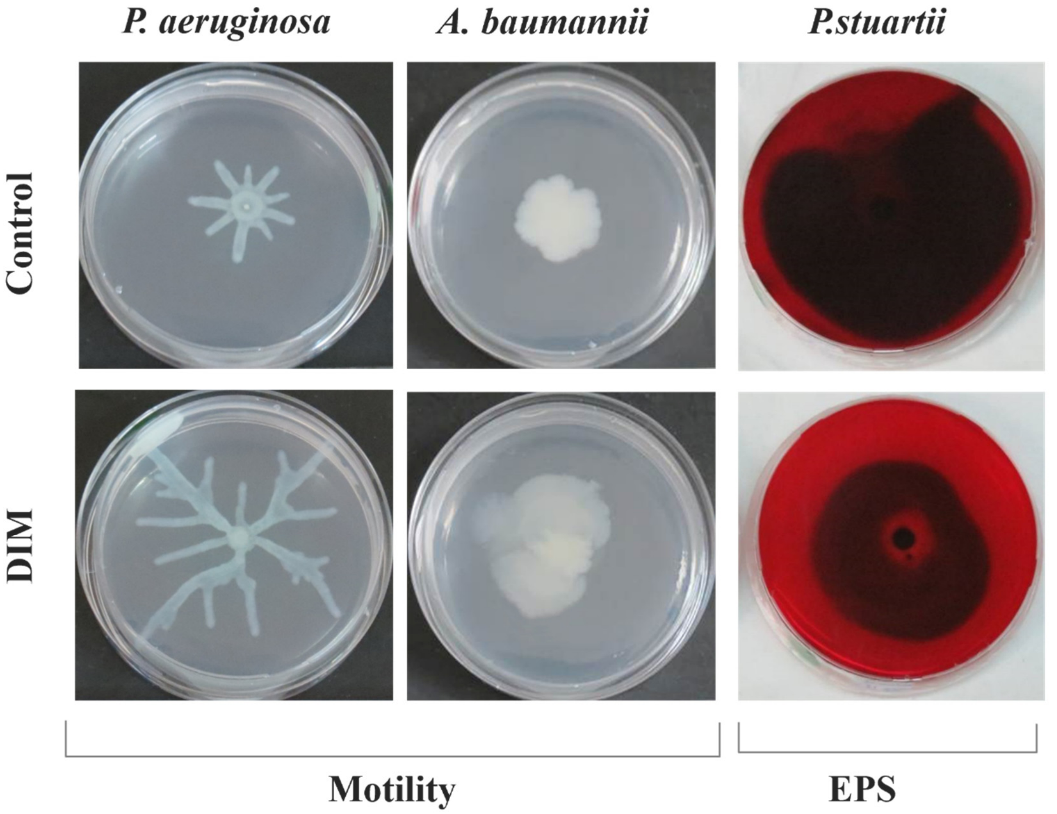

Figure S5. Qualitative assays of *P.aeruginosa* PAO1 and *A. baumannii* swarming motility. Each bacterium was grown on a 15-mm Petri dish in the presence of 100 µM of DIM or an equivalent amount of DMSO for control (out of five repeats). Radius of colony expansion indicates level of motility. Representative images (far right panel) from among three repeats of the Congo red plate qualitative assay of EPS production by *P. stuartii* show the black pigmentation that indicates secreted glucose-rich exopolysaccharides with or without 100 µM DIM.

Table S1. Primer sequences

Primer	Forward primer (5′–3′)	Reverse primer (5′–3′)	Tm (ºC)	GC (%)	Product length (bp)
proC	GGCGTATTTCTTCCTGCTGA	CCTGCTCCACTAGTGCTTCG	60	50.60	236
pilA	GTTGCGATCATCGGTATCCT	GTTTCGGTCGCAG-TAGAAGC	60	50.55	155
pilG	AATCCGACGGTTT-GAAAGTG	AACGCGCTGTTGTTCTTGAT	60	45.44	226
fliA	GGAAGACAC-CAGCCTCAGTC	CCGATCTCCTTGAGGTT-GAG	60	60.55	156
fliG	GATCCTGGAGAAGCAG-TTCG	TGTCGAAGAC-GAACATCAGG	60	55.50	185
PA4352	ATTCGGGGATCAAGGAA-GAC	GATCACGTTGATCGGCAAG	60	50.52	152
lasA	ATGGAC-CAGATCCAGGTGAG	CGTTGTCGTAGTTGCTGGTG	60	55.55	217
rhlA	CGAGGTCAATCAC-CTGGTCT	GACGGTCTCGTTGAG-CAGAT	60	55.55	208
phzM	TGCTGCGCGTAATTTGATAC	AGATCTCGAAGGCCACCAG	60	45.57	191

Table S2. Minimal inhibitory concentrations (MICs) of six antibiotics in µg/ml against *P. aeruginosa PAOmucA22*

	Control	DIM (50 µM)	% Susceptibility
Tobramycin	0.25	0.125	50
Colistin	1	0.75	25
Gentamicin	1.3	0.75	42
Ciprofloxacin	0.25	0.25	0
Azithromycin	7	5	29
Levofloxacin	5	3.5	30

Low MIC — High MIC

Bacterial colonies growing on agar have characteristics of planktonically growing cells and not of cells growing in a biofilm [1]. Therefore, to evaluate the most efficient antibiotic treatment in the biofilm experimental set-up, mucoid strain *PAOmucA22* which mimics aspects of enhanced resistance of biofilms was employed in the MIC assay. Mucoid biofilms are clinically the most pathogenic and the most resistant to antibiotics and attacks by the host immune system. The efficacy of using tobramycin in the combined treatment was validated with MIC tests of the antibiotics widely administered to pulmonary infection patients to increase the odds of obtaining higher biofilm eradication efficiency in the present study.

1. Mikkelsen, H.; Duck, Z.; Lilley, K.S.; Welch, M. Interrelationships between colonies, biofilms, and planktonic cells of Pseudomonas aeruginosa. *J. Bacteriol.* **2007**, *189*, 2411–2416. doi:10.1128/JB.01687-06
